# Benzyl *N*-(3-chloro-4-fluoro­phen­yl)carbamate

**DOI:** 10.1107/S1600536811012608

**Published:** 2011-04-16

**Authors:** Manavendra K. Singh, Alka Agarwal, Satish K. Awasthi

**Affiliations:** a Department of Medicinal Chemistry, Institute of Medical Sciences, Banaras Hindu University, Varanasi 225 001, India; b Chemical Biology Laboratory, Department of Chemistry, University of Delhi, Delhi 110 007, India

## Abstract

The title compound, C_14_H_11_ClFNO_2_, the phenyl ring (*A*), the chloro­fluoro­phenyl ring (*B*) and the central ketone O/C/O group (*C*) are not coplanar, with dihedral angles *B*/*C* = 31.6 (2), *A*/*B* = 21.3 (2) and *A*/*C* = 50.1 (2)°. The crystal packing is stabilized by N—H⋯O and C—H⋯O inter­actions.

## Related literature

For the bioactivity of nitro­gen-containing heterocyclic compounds, see: Xuan *et al.* (2001 ▶). For applications of anilines, see: Bickoff *et al.*(1952 ▶); Riegel & Kent (2007 ▶); Kahl *et al.* (2007 ▶). For our ongoing research on the anti­microbial activity of heterocyclic mol­ecules, see: Awasthi, Mishra, Dixit *et al.* (2009 ▶); Awasthi, Mishra, Kumar *et al.* (2009 ▶); Mishra *et al.* (2008 ▶). For the synthesis, see: Brickner *et al.* (1996 ▶).
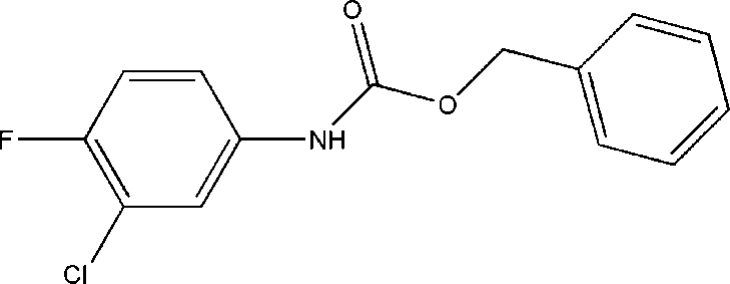


## Experimental

### 

#### Crystal data

C_14_H_11_ClFNO_2_
*M* *_r_* = 279.69
Orthorhombic, 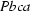

*a* = 10.4695 (16) Å
*b* = 9.0346 (11) Å
*c* = 28.361 (3) Å
*V* = 2682.6 (6) Å^3^
*Z* = 8
Cu *K*α radiation
μ = 2.62 mm^−1^
*T* = 293 K
0.40 × 0.39 × 0.38 mm

#### Data collection

Oxford Diffraction Xcalibur Sapphire3 diffractometer
Absorption correction: multi-scan (*CrysAlis PRO*; Oxford Diffraction, 2009 ▶) *T*_min_ = 0.668, *T*_max_ = 1.000
11782 measured reflections
2674 independent reflections
1547 reflections with *I* > 2σ(*I*)
*R*_int_ = 0.063

#### Refinement

*R*[*F* ^2^ > 2σ(*F* ^2^)] = 0.070
*wR*(*F* ^2^) = 0.234
*S* = 1.01
2674 reflections
184 parameters
H atoms treated by a mixture of independent and constrained refinement
Δρ_max_ = 0.26 e Å^−3^
Δρ_min_ = −0.48 e Å^−3^

### 

Data collection: *CrysAlis PRO* (Oxford Diffraction, 2009 ▶); cell refinement: *CrysAlis PRO*; data reduction: *CrysAlis PRO*; program(s) used to solve structure: *SHELXS97* (Sheldrick, 2008 ▶); program(s) used to refine structure: *SHELXL97* (Sheldrick, 2008 ▶); molecular graphics: *Mercury* (Macrae *et al.*, 2006 ▶); software used to prepare material for publication: *publCIF* (Westrip, 2010 ▶).

## Supplementary Material

Crystal structure: contains datablocks I, global. DOI: 10.1107/S1600536811012608/zj2005sup1.cif

Structure factors: contains datablocks I. DOI: 10.1107/S1600536811012608/zj2005Isup2.hkl

Additional supplementary materials:  crystallographic information; 3D view; checkCIF report

## Figures and Tables

**Table 1:** Hydrogen-bond geometry (Å, °)

*D*—H⋯*A*	*D*—H	H⋯*A*	*D*⋯*A*	*D*—H⋯*A*
N1—H1⋯O1^i^	0.83 (4)	2.09 (4)	2.906 (4)	164 (4)
C2—H2⋯O1^i^	0.93	2.67	3.414 (4)	138

Symmetry code: (i) 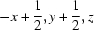.

**Fig. 1.**
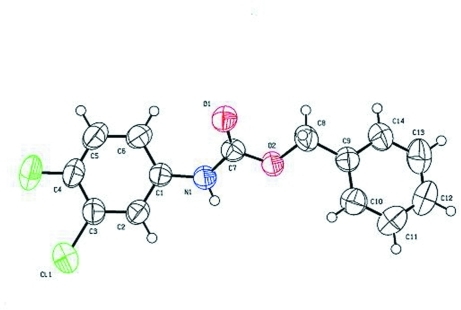
ORTEP view of the molecule with thermal ellipsoids drawn at 50% probability level Color code: White: C; red: O; blue: N; white: H; Green: Cl; Green: F.

**Fig. 2.**
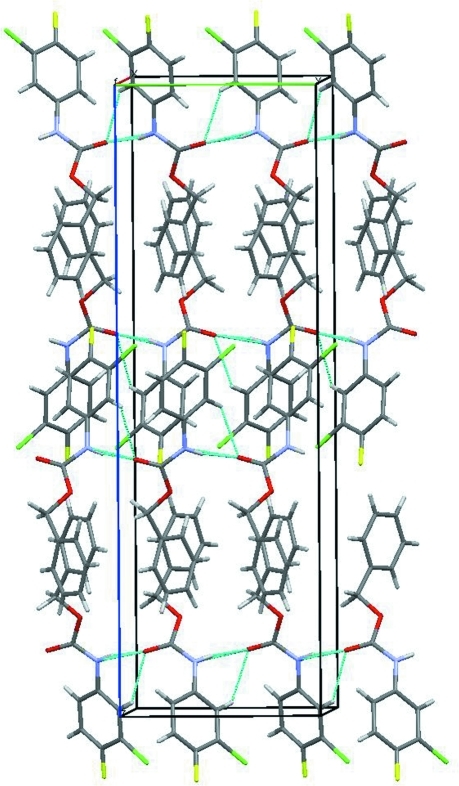
Packing diagram of molecule viewed through a plane showing Intermolecular hydrogen bonding in the molecule.

**Fig. 3.**
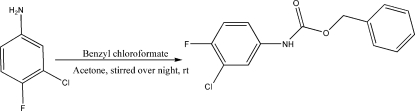
The formation of the title compound.

## supplementary crystallographic information

### Comment

Heterocyclic compounds containing nitrogen, oxygen, sulfur, *etc*. are well known for their antiviral, antimicrobial activities. Nitrogen containing heterocyclic compounds are unique due to the oxidation of nitrogen which is key factor for bioactivity of their scaffolds (Xuan *et al.*, 2001). Moreover, substituted anilines are widely used as intermediate in many organic synthesis as well as in many modern drugs. In aniline, the nitrogen atom is bonded to sp2 hybridized carbon atom. Further, the unshared electron pair on nitrogen atom of aniline can interact with the delocalized pi orbital of the nucleus and the aniline molecule is thus stabilized with respect to the anilinium cation. Here, the acceptance of a proton by aniline is energetically unfavorable. Therefore, it functions as a base with the utmost reluctance (p*K*_a_ = 4.62, compared with cyclohexylamine, p*K*_a_ = 10.68). The base weakening effect is naturally more pronounced when further phenyl groups are introduced on the nitrogen atom, thus diphenylamine, is an extremely weak base (p*K*_a_ = 0.8), while triphenylamine is not basic for all practical purpose. Further, aniline is widely used for synthesis of methylene diphenyl diisocynate (MDI). They are also used as rubber processing chemicals, herbicides, dyes and pigments (Riegel & Kent, 2007). Aniline derivatives such as phenylenediamine and diphenylamine are used as antioxidants (Bickoff *et al.*, 1952). Aniline is also used in the dye industry as a precursor to indigo, the blue of blue jeans (Kahl *et al.*, 2007). As part of our ongoing research on antimicrobial activities of some heterocyclic molecules (Awasthi, Mishra, Dixit *et al.*, 2009; Awasthi, Mishra, Kumar *et al.*, 2009; Mishra *et al.*, 2008), we report here the crystal structure of (3-chloro-4-fluro-phenyl)-carbamic acid benzyl ester (Figure 1). The crystal structure of molecule is stabilized by intermolecular hydrogen bonding and intermolecular interactions between N—H···O and C—H···O respectively as seen in Table 1, Figure 3. Considering C1—C6 of phenyl ring as plane 1 (PL1), central ketonic function O1C7O2 as plane 2 (PL2), and benzyl ring C8—C14 as plane 3 (PL3), the dihedral angels between planes PL 1 and PL2, PL1 and PL3, PL2 and PL3 are 31.65, 21.34, 50.13 respectively, suggests that the molecule is non-planar. The arrangement of molecules and its hydrogen bonding in the crystal can be seen in packing diagram (Figure 3).

### Experimental

The synthesis of title compound was achieved by published procedure (Brickner, *et al.*, 1996). Briefly, to a solution of 3-chloro-4-fluroaniline (1.0 g, 6.87 mmol) in acetone (25 ml) and water (12.5 ml) at 0¯C were added (1.18 g, 8.55 mmol) of sodium bicarbonate and then (1.01 ml, 7.08 mmol) of benzyl chloroformate over 6 min *via* syringe. The reaction mixture was stirred over night and then poured on ice water and filtered the solid and washed thoroughly with water. The product was recrystallized from dichloromethane. After several days leaving at room temperature, transparent white crystals were obtained by slow evaporation from dichloromethane at 6¯C. Yield = 1.64 g (85%), MS (Macromass G) m/*z* = 279.5 (*M*+), Rf 0.57 (98:2, CH2Cl2: MeOH) m.p. 60¯C, Elemental analysis (Perkin–Elmer 240 C elemental analyzer) Calculated for: C14H11O2NClF (%) C– 60.1, H– 3.9, O-11.5, N-5.0, Cl-12.7, F-6.8 found C-60.0, H-4.0, O– 11.0, N-4.8, Cl-12.6, F-7.1 ^1^H-NMR (CDCl3)- 7.56–7.55 (m, 1 H, H2), 7.02- 6.97 (m, 5H, ArH), 7.20–7.15 (m,1*H*, H6), 7.08–7.02(m, 1H, H5), 6.66 (s,1*H*, NH), 5.19 (s, 2H, CH2); ^13^ C-NMR (CDCl3): 155.97, 153.19, 135.66, 134.37, 128.65–128.35,121.33, 120.85,118.28,116.78,67.32

### Refinement

All H atoms were located from difference Fourier map (range of C—H = 0.93 - 1.08 Å,and N–H = 0.83 Å) allowed to refine freely

### Crystal data

**Table:**

C_14_H_11_ClFNO_2_	*F*(000) = 1152
*M**_r_* = 279.69	*D*_x_ = 1.385 Mg m^−^^3^
Orthorhombic, *P**b**c**a*	Cu *K*α radiation, λ = 1.54184 Å
Hall symbol: -P 2ac 2ab	Cell parameters from 1370 reflections
*a* = 10.4695 (16) Å	θ = 3.1–74.8°
*b* = 9.0346 (11) Å	µ = 2.62 mm^−^^1^
*c* = 28.361 (3) Å	*T* = 293 K
*V* = 2682.6 (6) Å^3^	Block, white
*Z* = 8	0.40 × 0.39 × 0.38 mm

### Data collection

**Table:**

Oxford Diffraction Xcalibur Sapphire3 diffractometer	2674 independent reflections
Radiation source: fine-focus sealed tube	1547 reflections with *I* > 2σ(*I*)
graphite	*R*_int_ = 0.063
ω scans	θ_max_ = 74.9°, θ_min_ = 3.1°
Absorption correction: multi-scan (*CrysAlis PRO*; Oxford Diffraction, 2009)	*h* = −12→11
*T*_min_ = 0.668, *T*_max_ = 1.000	*k* = −11→11
11782 measured reflections	*l* = −24→35

### Refinement

**Table:**

Refinement on *F*^2^	Primary atom site location: structure-invariant direct methods
Least-squares matrix: full	Secondary atom site location: difference Fourier map
*R*[*F*^2^ > 2σ(*F*^2^)] = 0.070	Hydrogen site location: inferred from neighbouring sites
*wR*(*F*^2^) = 0.234	H atoms treated by a mixture of independent and constrained refinement
*S* = 1.01	*w* = 1/[σ^2^(*F*_o_^2^) + (0.1301*P*)^2^] where *P* = (*F*_o_^2^ + 2*F*_c_^2^)/3
2674 reflections	(Δ/σ)_max_ = 0.050
184 parameters	Δρ_max_ = 0.26 e Å^−^^3^
0 restraints	Δρ_min_ = −0.48 e Å^−^^3^

### Special details

**Table:**

Geometry. All e.s.d.'s (except the e.s.d. in the dihedral angle between two l.s. planes) are estimated using the full covariance matrix. The cell e.s.d.'s are taken into account individually in the estimation of e.s.d.'s in distances, angles and torsion angles; correlations between e.s.d.'s in cell parameters are only used when they are defined by crystal symmetry. An approximate (isotropic) treatment of cell e.s.d.'s is used for estimating e.s.d.'s involving l.s. planes.
Refinement. Refinement of *F*^2^ against ALL reflections. The weighted *R*-factor *wR* and goodness of fit *S* are based on *F*^2^, conventional *R*-factors *R* are based on *F*, with *F* set to zero for negative *F*^2^. The threshold expression of *F*^2^ > σ(*F*^2^) is used only for calculating *R*-factors(gt) *etc*. and is not relevant to the choice of reflections for refinement. *R*-factors based on *F*^2^ are statistically about twice as large as those based on *F*, and *R*- factors based on ALL data will be even larger.

### Fractional atomic coordinates and isotropic or equivalent isotropic displacement parameters (Å^2^)

**Table:**

	*x*	*y*	*z*	*U*_iso_*/*U*_eq_	
F	0.4879 (3)	−0.1647 (3)	0.39260 (9)	0.1137 (11)	
H1	0.292 (4)	−0.103 (5)	0.5867 (14)	0.078 (13)*	
H8B	0.179 (4)	−0.457 (5)	0.6637 (16)	0.095 (14)*	
H8A	0.322 (5)	−0.426 (5)	0.6868 (15)	0.090 (15)*	
Cl1	0.30242 (15)	0.06343 (15)	0.41668 (4)	0.1034 (5)	
O1	0.3368 (3)	−0.4275 (2)	0.59477 (8)	0.0683 (7)	
N1	0.3282 (3)	−0.1806 (3)	0.57783 (10)	0.0619 (8)	
O2	0.2655 (3)	−0.2593 (2)	0.64774 (8)	0.0616 (7)	
C1	0.3700 (3)	−0.1824 (3)	0.53060 (11)	0.0542 (8)	
C2	0.3225 (4)	−0.0749 (4)	0.50081 (12)	0.0619 (9)	
H2	0.2637	−0.0063	0.5120	0.074*	
C7	0.3126 (3)	−0.3012 (3)	0.60581 (12)	0.0544 (8)	
C9	0.1806 (4)	−0.3126 (4)	0.72378 (12)	0.0612 (9)	
C3	0.3623 (4)	−0.0687 (4)	0.45406 (12)	0.0669 (10)	
C4	0.4493 (4)	−0.1710 (5)	0.43833 (14)	0.0741 (11)	
C14	0.2263 (5)	−0.3472 (5)	0.76831 (13)	0.0799 (12)	
H14	0.2967	−0.4092	0.7715	0.096*	
C6	0.4595 (4)	−0.2837 (4)	0.51389 (14)	0.0691 (10)	
H6	0.4934	−0.3551	0.5339	0.083*	
C5	0.4974 (4)	−0.2771 (5)	0.46710 (16)	0.0777 (12)	
H5	0.5559	−0.3454	0.4554	0.093*	
C8	0.2409 (6)	−0.3783 (4)	0.68092 (14)	0.0727 (12)	
C12	0.0646 (5)	−0.1989 (6)	0.80410 (16)	0.0924 (15)	
H12	0.0258	−0.1606	0.8310	0.111*	
C10	0.0767 (4)	−0.2206 (4)	0.72049 (15)	0.0736 (11)	
H10	0.0448	−0.1956	0.6909	0.088*	
C11	0.0188 (5)	−0.1646 (5)	0.76045 (16)	0.0879 (13)	
H11	−0.0519	−0.1031	0.7576	0.106*	
C13	0.1679 (6)	−0.2899 (6)	0.80796 (16)	0.1000 (16)	
H13	0.1995	−0.3137	0.8377	0.120*	

### Atomic displacement parameters (Å^2^)

**Table:**

	*U*^11^	*U*^22^	*U*^33^	*U*^12^	*U*^13^	*U*^23^
F	0.134 (3)	0.132 (2)	0.0748 (16)	0.013 (2)	0.0481 (16)	−0.0009 (15)
Cl1	0.1209 (11)	0.1169 (11)	0.0724 (7)	0.0226 (8)	0.0116 (6)	0.0251 (6)
O1	0.092 (2)	0.0425 (13)	0.0708 (15)	−0.0037 (12)	0.0111 (13)	−0.0078 (10)
N1	0.084 (2)	0.0432 (15)	0.0582 (16)	0.0042 (14)	0.0088 (15)	−0.0015 (12)
O2	0.0882 (18)	0.0455 (12)	0.0511 (12)	0.0016 (11)	0.0080 (12)	−0.0006 (9)
C1	0.060 (2)	0.0470 (17)	0.0557 (17)	−0.0024 (14)	0.0038 (15)	−0.0008 (13)
C2	0.064 (2)	0.058 (2)	0.0635 (19)	0.0014 (16)	0.0106 (16)	−0.0055 (16)
C7	0.062 (2)	0.0425 (16)	0.0589 (17)	−0.0012 (14)	−0.0042 (15)	−0.0039 (14)
C9	0.071 (2)	0.0527 (19)	0.0600 (18)	−0.0037 (16)	0.0015 (17)	−0.0011 (15)
C3	0.071 (2)	0.071 (2)	0.0585 (19)	−0.0025 (18)	0.0092 (17)	0.0025 (17)
C4	0.077 (3)	0.083 (3)	0.063 (2)	−0.001 (2)	0.0237 (19)	−0.006 (2)
C14	0.086 (3)	0.094 (3)	0.060 (2)	0.009 (2)	−0.001 (2)	0.009 (2)
C6	0.066 (2)	0.060 (2)	0.081 (2)	0.0054 (17)	0.0158 (19)	−0.0007 (18)
C5	0.076 (3)	0.072 (2)	0.085 (3)	0.005 (2)	0.029 (2)	−0.009 (2)
C8	0.107 (4)	0.050 (2)	0.061 (2)	0.003 (2)	0.012 (2)	0.0048 (16)
C12	0.085 (3)	0.115 (4)	0.077 (3)	−0.010 (3)	0.025 (2)	−0.021 (3)
C10	0.079 (3)	0.071 (3)	0.071 (2)	−0.001 (2)	−0.006 (2)	−0.0047 (18)
C11	0.074 (3)	0.095 (3)	0.094 (3)	0.008 (2)	0.008 (2)	−0.020 (3)
C13	0.115 (4)	0.125 (4)	0.060 (2)	−0.006 (3)	0.003 (3)	−0.007 (2)

### Geometric parameters (Å, °)

**Table:**

F—C4	1.360 (4)	C4—C5	1.356 (6)
Cl1—C3	1.715 (4)	C14—C13	1.381 (6)
O1—C7	1.210 (4)	C14—H14	0.9300
N1—C7	1.358 (4)	C6—C5	1.387 (6)
N1—C1	1.409 (4)	C6—H6	0.9300
N1—H1	0.83 (4)	C5—H5	0.9300
O2—C7	1.342 (4)	C8—H8B	1.08 (5)
O2—C8	1.452 (4)	C8—H8A	0.97 (5)
C1—C6	1.393 (5)	C12—C13	1.363 (7)
C1—C2	1.380 (5)	C12—C11	1.363 (6)
C2—C3	1.391 (4)	C12—H12	0.9300
C2—H2	0.9300	C10—C11	1.381 (6)
C9—C10	1.372 (6)	C10—H10	0.9300
C9—C14	1.386 (5)	C11—H11	0.9300
C9—C8	1.492 (5)	C13—H13	0.9300
C3—C4	1.372 (5)		
			
C7—N1—C1	125.7 (3)	C5—C6—C1	119.3 (4)
C7—N1—H1	116 (3)	C5—C6—H6	120.3
C1—N1—H1	116 (3)	C1—C6—H6	120.3
C7—O2—C8	115.5 (3)	C4—C5—C6	120.0 (4)
C6—C1—C2	119.7 (3)	C4—C5—H5	120.0
C6—C1—N1	122.7 (3)	C6—C5—H5	120.0
C2—C1—N1	117.5 (3)	O2—C8—C9	108.0 (3)
C3—C2—C1	120.3 (3)	O2—C8—H8B	108 (2)
C3—C2—H2	119.8	C9—C8—H8B	112 (2)
C1—C2—H2	119.8	O2—C8—H8A	107 (3)
O1—C7—O2	124.9 (3)	C9—C8—H8A	114 (3)
O1—C7—N1	125.5 (3)	H8B—C8—H8A	108 (4)
O2—C7—N1	109.6 (3)	C13—C12—C11	119.3 (4)
C10—C9—C14	118.2 (4)	C13—C12—H12	120.4
C10—C9—C8	121.4 (4)	C11—C12—H12	120.4
C14—C9—C8	120.4 (4)	C11—C10—C9	120.9 (4)
C4—C3—C2	118.8 (4)	C11—C10—H10	119.5
C4—C3—Cl1	120.7 (3)	C9—C10—H10	119.5
C2—C3—Cl1	120.5 (3)	C12—C11—C10	120.5 (5)
C5—C4—F	119.5 (4)	C12—C11—H11	119.7
C5—C4—C3	121.8 (4)	C10—C11—H11	119.7
F—C4—C3	118.7 (4)	C12—C13—C14	120.8 (5)
C9—C14—C13	120.3 (5)	C12—C13—H13	119.6
C9—C14—H14	119.9	C14—C13—H13	119.6
C13—C14—H14	119.9		
			
C7—N1—C1—C6	−34.6 (6)	C8—C9—C14—C13	−178.0 (5)
C7—N1—C1—C2	147.9 (4)	C2—C1—C6—C5	−1.5 (6)
C6—C1—C2—C3	0.9 (6)	N1—C1—C6—C5	−179.0 (4)
N1—C1—C2—C3	178.5 (3)	F—C4—C5—C6	179.5 (4)
C8—O2—C7—O1	−1.2 (6)	C3—C4—C5—C6	−0.4 (7)
C8—O2—C7—N1	178.5 (4)	C1—C6—C5—C4	1.2 (7)
C1—N1—C7—O1	2.8 (6)	C7—O2—C8—C9	−176.0 (3)
C1—N1—C7—O2	−176.9 (3)	C10—C9—C8—O2	50.9 (6)
C1—C2—C3—C4	−0.1 (6)	C14—C9—C8—O2	−131.2 (4)
C1—C2—C3—Cl1	179.3 (3)	C14—C9—C10—C11	−0.3 (6)
C2—C3—C4—C5	−0.2 (6)	C8—C9—C10—C11	177.7 (4)
Cl1—C3—C4—C5	−179.6 (4)	C13—C12—C11—C10	−0.4 (8)
C2—C3—C4—F	179.9 (4)	C9—C10—C11—C12	0.5 (7)
Cl1—C3—C4—F	0.5 (6)	C11—C12—C13—C14	0.1 (8)
C10—C9—C14—C13	0.0 (7)	C9—C14—C13—C12	0.1 (8)

### Hydrogen-bond geometry (Å, °)

**Table:**

*D*—H···*A*	*D*—H	H···*A*	*D*···*A*	*D*—H···*A*
N1—H1···O1^i^	0.83 (4)	2.09 (4)	2.906 (4)	164 (4)
C2—H2···O1^i^	0.93	2.67	3.414 (4)	138

Symmetry codes: (i) −*x*+1/2, *y*+1/2, *z*.
